# Genome-Wide Analysis of the MADS-Box Transcription Factor Family in *Solanum lycopersicum*

**DOI:** 10.3390/ijms20122961

**Published:** 2019-06-18

**Authors:** Yunshu Wang, Jianling Zhang, Zongli Hu, Xuhu Guo, Shibing Tian, Guoping Chen

**Affiliations:** 1 Laboratory of molecular biology of tomato, Bioengineering College, Chongqing University, Chongqing 400044, China; wangyunshu@cqu.edu.cn (Y.W.); zhangjianling0520@126.com (J.Z.); huzongli71@163.com (Z.H.); 20131901005@cqu.edu.cn (X.G.); 2 The Institute of Vegetable Research, Chongqing Academy of Agricultural Sciences, Chongqing 401329, China; tiansbing@aliyun.com

**Keywords:** tomato, MADS-box, genome-wide analysis, floral organ, fruit development

## Abstract

MADS-box family genes encode transcription factors that are involved in multiple developmental processes in plants, especially in floral organ specification, fruit development, and ripening. However, a comprehensive analysis of tomato MADS-box family genes, which is an important model plant to study flower fruit development and ripening, remains obscure. To gain insight into the MADS-box genes in tomato, 131 tomato MADS-box genes were identified. These genes could be divided into five groups (Mα, Mβ, Mγ, Mδ, and MIKC) and were found to be located on all 12 chromosomes. We further analyzed the phylogenetic relationships among Arabidopsis and tomato, as well as the protein motif structure and exon–intron organization, to better understand the tomato MADS-box gene family. Additionally, owing to the role of MADS-box genes in floral organ identification and fruit development, the constitutive expression patterns of MADS-box genes at different stages in tomato development were identified. We analyzed 15 tomato MADS-box genes involved in floral organ identification and five tomato MADS-box genes related to fruit development by qRT-PCR. Collectively, our study provides a comprehensive and systematic analysis of the tomato MADS-box genes and would be valuable for the further functional characterization of some important members of the MADS-box gene family.

## 1. Introduction

The MADS-box family genes encode transcription factors (TFs), which are widely distributed in eukaryotes and play fundamental roles in diverse biological functions [1]. The name MADS-box is derived from the initials of four transcription factors that were first discovered of this family: MINICHROMOSOME MAINTENANCE 1 (MCM1), AGAMOUS (AG), DEFICIENS (DEF), and SERUM RESPONSE FACTOR (SRF) [2]. Their N-terminal contains a highly conserved DNA-binding MADS-domain containing 56–60 amino acids [2]. Thus, a protein encoding the MADS-domain is referred to as a MADS-box protein. MADS-box genes are divided into two groups (type I and type II) throughout the eukaryotes [3]. Type I MADS-box transcription factors can be further classified into four subclasses (Mα, Mβ, Mγ, and Mδ) in view of the M domain of the encoded protein, while only a few type I genes have been characterized for their biological function [4]. Type II MADS-box transcription factors include the Myocyte Enhancer Factor 2-like (MEF2-like) group from animals and yeast and the plant-specific MIKC-type group. The name of the MIKC-type protein is derived from their four characteristic domains: MADS-box (M), intervening (I), keratin-like (K), and C-terminal (C) [5]. The MIKC type has been further subdivided into MIKC^C^ and MIKC* types based on phylogenetic relationships and structural features [4]. The functions of the type II genes, especially the MIKC^C^ type MADS-box genes in plants, have been reported in more detail. To date, 39 MIKC^C^ type MADS-box genes were found in *Arabidopsis thaliana* [6], and 38 MIKC^C^ type MADS-box genes were found in rice [7].

MADS-box genes are known to play important roles in different aspects of plants growth and development. The MADS-box genes *AGAMOUS* (*AG*) of *Arabidopsis thaliana* [8] as well as *DEFICIENS* (*DEF*) of *Antirrhinum majus* [2] were found to regulate floral organ formation two decades ago. The MADS-box genes were thought to be the main participants in floral organ specificity. With the analysis of the specification of floral organ identity determination, a genetic model (ABC model) was proposed in which the different steps of floral development were determined by three classes of genes (A, B, and C) [9]. Class A genes are necessary for the formation of the sepal. The class A genes, together with class B genes, determine the development of petals. The combination of class B and C genes is necessary for stamen identity, and class C genes function alone to form carpels [10]. However, there are many phenomena that this model cannot explain. For example, the constitutive co-expression of the B class genes *PISTILLATA* (*PI*) and *APETALA3* (*AP3*) in *Arabidopsis thaliana* [11], does not change the identity of vegetative organs. Recent studies found that the ABC model is necessary but not sufficient to provide the floral organ identity function. Therefore, the model was advanced by subdivision into five different classes (A–E). Class D genes specify ovule development, while class E genes are necessary for the specification of petals, stamens, and carpels. In addition, these five classes of genes are mainly MADS-box genes [12]. In *Arabidopsis thaliana*, *APETALA1* (*AP1*) is a typical class A gene [13], *APETALA3* (*AP3*) and *PISTILLATA* (*PI*) belong to the class B genes [14], and *AGAMOUS* (*AG*) is a representative gene with class C function [15]. The *SEPALLATA* (*SEP*) genes are class E genes and include *SEP1*, *SEP2*, *SEP3,* and *SEP4* in *Arabidopsis thaliana* [16]. The class D gene was identified by the mutant phenotype of petunia, and the sequence similarity analysis demonstrated that the corresponding gene in Arabidopsis thaliana is *AGAMOUS-LIKE11* (*AGL11*) [17,18].

In addition, considerable evidence has revealed that MADS-box TFs share a potent effect on the regulation of fruit development and ripening [4]. There have been many studies on the function of *Solanum lycopersicum* MADS-box genes in fruit development and ripening. The tomato MADS-box gene *RIPENING INHIBITOR* (*RIN*) is an essential factor for fruit ripening that regulates many ripening-associated processes, including both ethylene-dependent and ethylene-independent ripening processes [19]. Moreover, the tomato MADS-box genes *AGAMOUSLIKE1* (*AGL1*) [20,21], *FRUITFULL1* (*FUL1*), and *FRUITFULL2* (*FUL2*) are Arabidopsis *SHATTERPROOF* (*SHP*) and *FRUITFUL* (*FULL*) homologues respectively [22,23], and their suppression results in a phenotype that is partially similar to the ripening-defective phenotype of *RIN* mutant fruits. In addition to crucial roles in the regulation of plant reproductive development, MADS-box genes have also been shown to take part in plant vegetative growth processes and some stress responses in different plants such as Arabidopsis [24], rice [7,25], wheat [26], and Chinese cabbage [27]. Thus, the MADS-box protein family is an important TFs family for plant growth and development that almost affects the whole process of plant growth and development, especially plant reproductive development.

The MADS-box family of model plant species has been widely studied, including snapdragon (*Antirrhinum majus*) [28], rice (*Oryza sativa)* [7], Chinese cabbage (*Brassica rapa*) [27], poplar (*Populus trichocarpa*) [29], bread wheat (*Triticum aestivumL*) [30], banana (*Musa acuminata*) [31], petunia (*Petunia hybrida*) [32], apple (*Malus domestica Borkh)* [33], and so on. Tomato (*Solanum lycopersicum*) is one of the most critical model plants for studying flower and fruit development, and the MADS-box genes of tomato were among the earliest to be investigated [34]. It has been reported that 24 tomato MIKC^C^-type MADS-box genes had been identified in 2006 [35]. However, there has been no report to date concerning systematic information for the tomato MADS-box gene family, and confusion in the names of these genes is problematic for future research.

To obtain a genome-wide analysis of the tomato MAD-box gene family, we sorted 131 MADS-box genes from tomato that are highly homologous to MADS-box proteins known in other plant species, analyzed their phylogenetic relationships, gene structure, and conserved motifs, determined their exon–intron organization, and predicted their chromosomal localization. Furthermore, we obtained the predictions of the expression pattern of these tomato MADS-box genes in order to predict their expression pattern. In addition, the expression pattern of some MADS-box genes related to tomato flower organ identity, fruit development, and ripening were determined by qPCR analyses in different stages of tomato development. These results provide details of the tomato MADS-box family and may be useful for more comprehensive investigations of tomato MADS-box gene family members.

## 2. Results

### 2.1. Identification of MADS-Box Genes in Tomato

To extensively identify tomato MADS-box genes, a set of 131 tomato MADS-box genes that are highly homologous to the MADS-box proteins reported in other plants was recovered using BLAST searches against the NCBI and SGN databases. Redundant sequences were removed. Since 35 tomato MADS-box genes have been officially named, the newly identified 96 tomato MADS-box genes were designated as *SlMADS2*–*SlMADS98* (Table 1). Additionally, the molecular characteristics of the 131 MADS-box genes in tomato were analyzed. The names of the tomato MADS-box genes, the locus/gene names of SGN, the length of amino acid sequence, the molecular weight, and the isoelectric point are shown in Table 1. The statistical results showed that the amino acid sequence length of the 131 predicted tomato MADS-box proteins varied from 54 (*SlMBP19*) to 417 (*SlMADS52*), the relative molecular mass ranged from 6224.26 Da (*SlMBP19*) to 47275.1 Da (*SlMADS52*), and the isoelectric point (pI) varied from 4.41 (*SlMADS70*) to 11.03 (*SlMADS77* and *SlMADS80*). The homologous genes of the tomato MADS-box family genes in Arabidopsis and the references for the study of the functions in homologous genes are shown in Table S1. In addition, the functional reports of several genes that have been studied previously were listed.

### 2.2. Classification and Phylogenetic Analysis of Tomato MADS-Box Genes

To study the phylogenetic relationships among MADS-box genes in tomato and Arabidopsis [6], a phylogenetic tree was drawn by the neighbor-joining (NJ) method using MEGA 5.02 (Figure S1A). Based on previous reports on Arabidopsis, the 131 tomato MADS-box genes were classified into two types: type I (81) and type II (50). Based on the phylogenetic tree, type I and type II tomato MADS-box genes were subdivided into more detailed subgroups. Then, type I could be further divided into four groups (Mα, Mβ, Mγ, and Mδ), while Type II (MIKC) could be further divided into MIKC* and MIKC^C^. The MIKC^C^-type genes included the *AP3*/*PI*, *SVP*, *AGL15*, *SEPALLATA* (*SEP*), *AGL6*, *AP1*, *FLOWERING LOCUS C* (*FLC*) *SOC1*, *AGAMOUS* (*AG*), *TM8,* and *DEFICIENS* (*DEF*)/*GLOBOSA* (*GLO*) subfamilies, similar to the MADS-box genes in other plants species. In addition, the phylogenetic tree of type I and type II MADS-box protein in tomato plants were constructed to identify the phylogenetic relationships of gene numbers in the two types of tomato MADS-box family more clearly (Figure S1B,C).

### 2.3. Conserved Motif and Gene Structure Analysis of Tomato MADS-Box Genes

**Figure 1:**
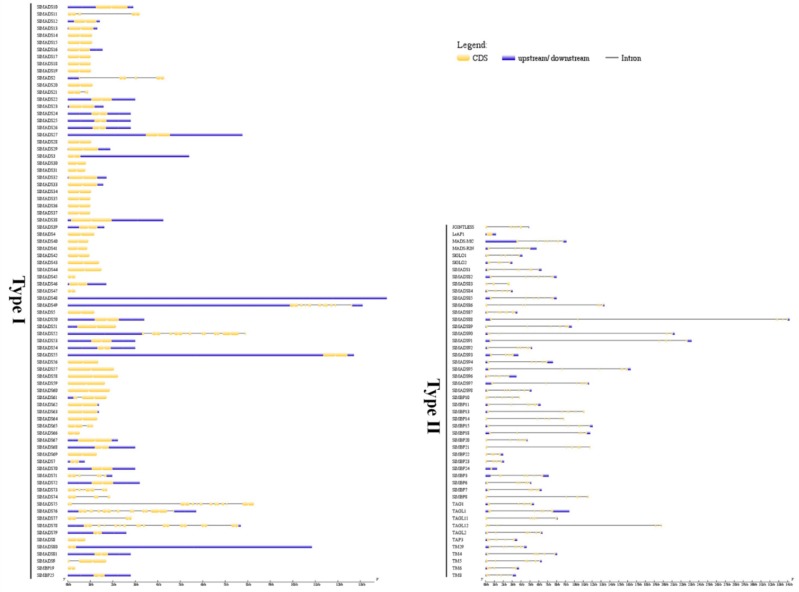
Gene structure analysis of MADS-box genes in tomato. The Gene Structure Display Server (GSDS) database was used to perform the exon–intron structure analyses. Lengths of exons and introns of each MADS-box gene were displayed proportionally. The blue boxes represent upstream/downstream, the yellow boxes represent exons, and the black lines represent introns.

The intron–exon organization was analyzed to investigate the structural diversity and evolution of the 131 tomato MADS-box genes. As shown in Figure 1, we obtained each gene’s intron/exon arrangement by comparing their CDS with their genomic sequences using the program Gene Structure Display Server (GSDS). The number of introns in tomato MADS-box genes ranged from one to 11. Similar to Arabidopsis, the distribution of introns in tomato was different in type I and type II genes [6]. In our study, we found that the Mα, Mβ, and Mγ groups of the type I genes usually had no introns or one intron, which might be explained by the diversity of the reverse-transcribed origin or the differences in acquisition or loss introns by the ancestors of these three groups of genes [6]. Based on the genomic data, the Mδ group of type I and the type II genes contained multiple introns. Among the Mδ clade and the type II genes, 52 of 56 (92.9%) genes had more than five introns. Additionally, the gene structures of closely related genes in tomato MADS-box genes were more similar, and the differences were only in the lengths of introns and exons. However, some close gene pairs showed different intron/exon arrangements. For instance, *SlMBP61* has one exon, whereas its close homologs *SlMBP51* and *SlMBP10* both have two, although their phylogenetic relationship displayed a high bootstrap value (Figure S1B).

**Figure 2:**
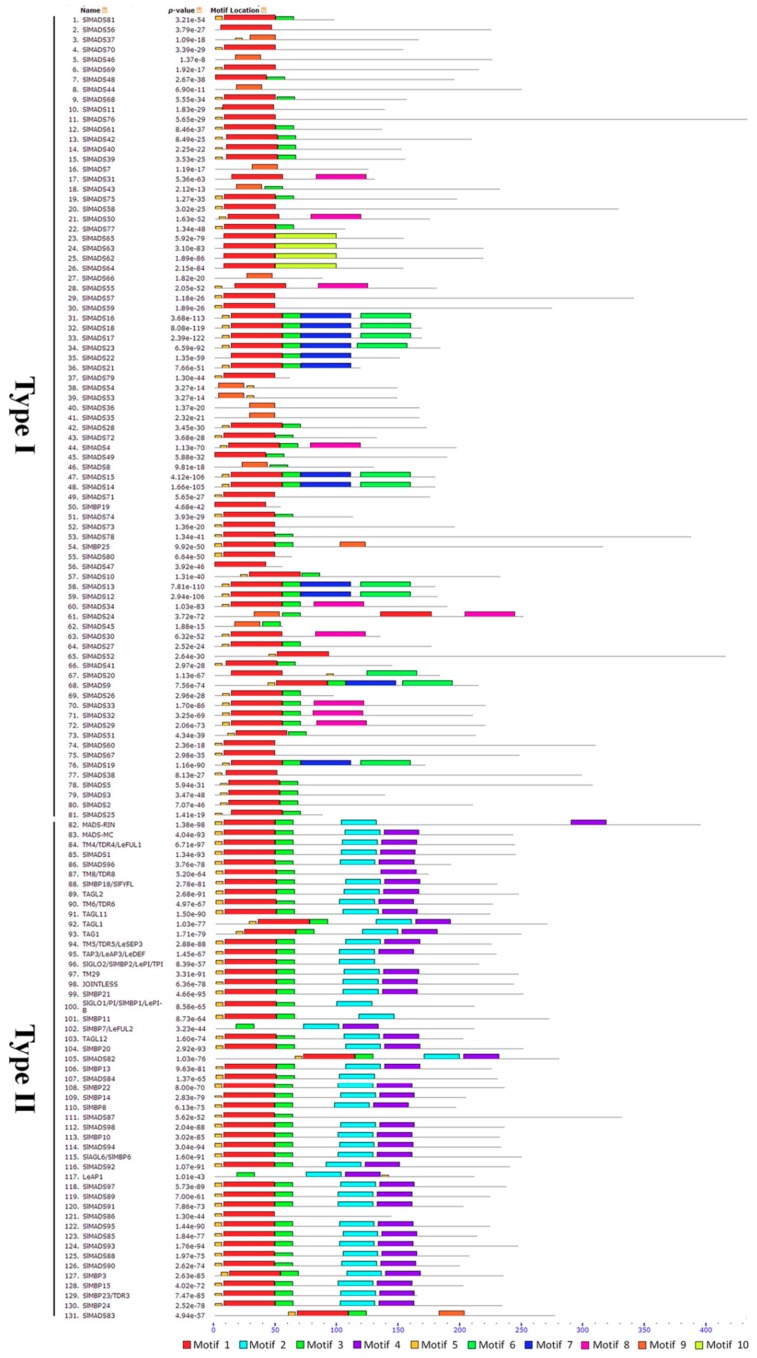
Conserved motif analyses of MADS-box genes in tomato. All the conserved motifs were identified by the Multiple EM for Motif Elicitation (MEME) database using the complete amino acid sequences of tomato MADS-box genes. Ten conserved different motifs were indicated by different colors. The details of motifs refer to the Supplementary Figure S2.

To better analyze conserved motifs in tomato MADS-box proteins, we constructed a conserved motif figure using the Multiple EM for Motif Elicitation (MEME) program and annotated them using SMART. A total of 10 conserved motifs, named 1 to 10, were identified (Figure 2). The details of the motifs are shown in Figure S2. As expected, the same types of genes tend to possess the same motifs. Motif 1—one of the most typical MADS-box domains—comprised 42 amino acids was found in the majority of tomato MADS-box proteins. Motif 3 was also conserved across most of the tomato MADS-box proteins, including type I and type II genes. Motifs 2 and 4 represent the K domain, which plays an important role in protein–protein interactions among MADS-box proteins, and they were found only in type II MADS-box proteins. Motif 2 was identified in almost all the type II proteins except for TM8/TDR8, SlMADS86, SlMADS87, and SlMADS83. In the type II proteins, a large number of proteins had motif 4, with seven exceptions (SlGLO2, SlGLO1, SlMBP11, SlMADS84, SlMADS86, SlMADS87, and SlMADS83). Motif 9 is also a MADS-box domain that is present in a small number of tomato MADS-box proteins. However, some motifs (6, 7, 8, and 10) were shown to be weakly conserved in tomato MADS-box proteins, and they were found only in type I MADS-box proteins.

### 2.4. Chromosomal Locations of Tomato MADS-Box Genes

According to physical genome annotation files that were obtained by using genomic sequences from the SGN and NCBI databases, 131 members of the MADS-box genes were located on all 12 tomato chromosomes, implying that the MADS-box transcription factor family may have multiple functions in tomato plants.

**Figure 3:**
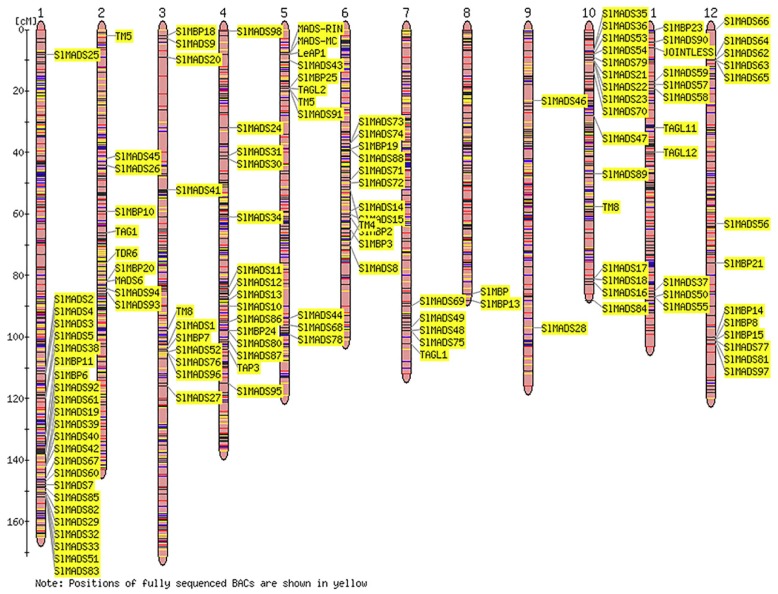
Chromosomal locations of tomato MADS-box genes. A total of 12 chromosomes of tomato were labeled with their names, chromosomes 1 to 12, which are indicated at the top of each bar. The position of tomato MADS-box genes on the chromosome was based on the Sol Genomics Network (SGN) and National Center for Biotechnology Information (NCBI) database and the Tomato-EXPEN 2000 was used to draw the physical map of the tomato MADS-box genes.

As shown in Figure 3, the tomato MADS-box genes are distributed unevenly on each chromosome. Chromosome 1 contains the most MADS-box genes (24), while chromosome 8 and 9 contain the fewest (two). Gene duplication events have a significant function in amplifying gene family numbers and genome complexity in eukaryotes [73,74]. The tandem amplification or segmental duplication of chromosomal regions can increase gene families. In this study, the results showed that chromosomes 1, 3, 4, 10, and 12 contain gene clusters or gene hotspots; in particular, chromosome 1 contains eight MADS-box genes within a short region. Additionally, we confirmed that internal chromosome duplication (tandem duplication) occurred in these genes.

### 2.5. Predictions of Expression Profiles of Tomato MADS-Box Genes in Different Organs

**Figure 4:**
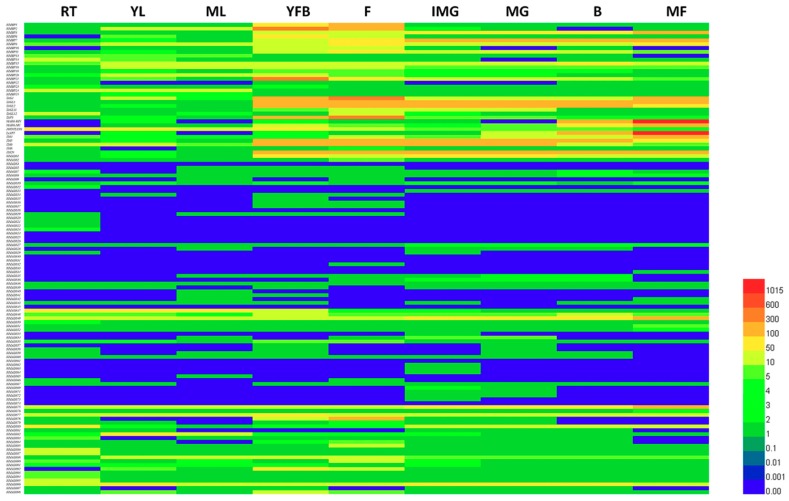
Heat map representation of tomato MADS-box genes in various tissues. The tissues included the whole root (Rt), young leaf (YL), mature leaves (ML), young flower buds (YFB), and fully open flowers (F), which were at the immature green (IMG), mature green (MG), break (B), and mature (MF) stages of fruit development and ripening. The bar at the bottom of the heat map represents relative gene expression values.

To investigate the tomato MADS-box genes expression patterns in different tissues of tomato plants, we analyzed tomato transcript expression (RNA-seq) data in nine different tomato tissues at different developmental stages. This included the expression in the whole root (RT), young leaf (YL), mature leaves (ML), young flower buds (YFB), fully open flowers (F), and at the immature green (IMG), mature green (MG), break (B), and mature (MF) stages of fruit development and ripening. These datasets were searched using the locus/gene names in SGN of 124 tomato MADS-box gene sequences, except for *SlMADS4*, *SlMADS11*, *SlMADS37*, *SlMADS44*, *SlMADS46*, *SlMADS56*, *SlMADS68*, *SlMADS70* and *SlMADS89*, which were not accurately found in TFGD. Then, we constructed a hierarchical clustering heat map using these datasets (Figure 4).

The expression profiles revealed that 117 genes were expressed in at least one tomato plant organ, while the other seven genes (*SlMADS24*, *SlMADS25*, *SlMADS26*, *SlMADS33*, *SlMADS45*, *SlMADS61*, and *SlMADS74*) were expressed at levels that were too low to be identified, or they had temporal and spatial specific expression patterns that showed no expression in the organs tested. Most tomato MADS-box genes displayed a broad expression range across all the organs and developmental stages, which is consistent with previous reports that the MADS-box genes may play multiple roles in plant growth and development [75,76]. However, some genes exhibited tissue-specific expression. For example, the expression of *SlMADS12*, *SlMADS20*, *SlMADS21*, *SlMADS22,* and *SlMADS23* were restricted in whole root, and the *SlMADS16, SlMADS17,* and *SlMADS132* transcripts were observed only during flower development. These results illustrate that these genes may be involved in the regulation of some biological process of the tomato root or in flower growth and development. Eight genes (*SlMBP2*, *SlMBP6*, *SlMBP10*, *SlMBP21*, *TAP3*, *SlMADS78*, *SlMADS92*, and *SlMADS98*) showed especially high expression in young flower buds (YFB) and fully open flowers (F), indicating that these genes may play important roles in floral organ development. We further discovered that most type II genes (*SlMBP3*, *SlMBP7*, *SlMBP11*, *SlMBP15*, *MADS-RIN*, *MADS-MC*, *TAGL1*, and *LeAP1*) were highly expressed during flower or fruit development; especially, the expression values of *MADS-RIN* and *MADS-MC* in fruits were more than 1000, suggesting that these genes may be associated with the reproductive growth of tomato. However, the expression of most type II genes showed no significant difference among tissues.

In short, these results indicate that the MADS-box genes had different expression levels in various tomato organs, and the predictions of the organ expression profiles of the tomato MADS-box gene family may provide insight for future studies on the functions of MADS-box genes in tomato plant growth and development.

### 2.6. The Critical Tomato MADS-Box Genes Involved in Floral Organ Development

Based on the phylogenetic analysis of the MADS-box genes that participated in floral organ development in petunia, which has been reported previously [32,77] (Figure S3), 15 tomato MADS-box genes that are possibly involved in floral organ development were screened out, including tow class A genes (*MADS-MC* and *SlMBP20*), four class B genes (*TAP3*, *TM6*, *SlMBP1,* and *SlMBP2*, two class C genes (*TAG1* and *TAGL1*), two class D genes (*SlMBP3* and *SlMBP22*), and five class E genes (*TAGL2*, *TM5*, *SlMADS1*, *SlMBP21,* and *SlMBP6*), as shown in Table S2.

According to the expression profile predictions shown in Figure 4, among the 15 MADS-box genes, nine genes (*MADS-MC*, *TAP3*, *TM6*, *SlMBP1*, *SlMBP2*, *TAG1*, *SlMBP22, SlMBP21,* and *SlMBP6*) were extremely highly expressed in flowers. However, *TAGL1*, *SlMBP3*, *TAGL2*, *TM5*, and *SlMADS1* were mainly expressed during the stages of fruit development and ripening, and the expression of *SlMBP20* was particularly high in leaves.

**Figure 5:**
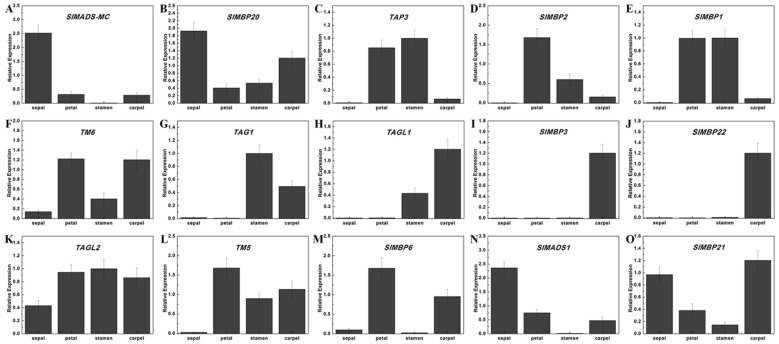
Expression profiles of floral organ identity genes in the four-whorl floral organs of the wild-type (WT) tomato plant for qPCR analysis. Se, sepal; Pe, petal; St, stamen; Ca, carpel. (A)–(O) Expression profiles of *SlMADS*-*MC*, SlMBP*21*, *TAP3*, *SlMBP2*, *SlMBP1*, *TM6*, *TAG1*, *TAGL1*, *SlMBP3*, *SlMBP22*, *TAGL2*, *TM5*, *SlMBP6*, *SlMADS1* and *SlMBP21*.Each value represents the mean ± SE of three technical replicates of a single biological sample. The *SlCAC* gene of tomato was used as the internal standard.

To further investigate the potential role of these MADS-box genes in floral organ development, a comparison of the expression patterns of 15 tomato MADS-box genes in four whorls of floral organs (sepal, petal, stamen, and carpel) were analyzed by qPCR. As shown in Figure 5A,B, the class A genes *MADS-MC* and *SlMBP20* were highly expressed in sepals. The expression levels of class B genes (*TAP3*, *SlMBP2,* and *SlMBP1)* were notably high in the petal and stamen (Figure 5C–E), whereas the transcription of *TM6* was found to be markedly higher in carpel and stamen compared with other floral organs (Figure 5F). Two class C genes, *TAG1* and *TAGL1*, were found to be mainly expressed in stamens and carpels (Figure 5G,H). Moreover, the class D genes, *SlMBP3* and *SlMBP22,* showed organ-specific expression patterns that were exclusively expressed in carpel (Figure 5I,J). The expression patterns of class E genes indicated that the expression of *TAGL2* and TM5 were significantly higher expressed in the petal, stamen, and carpel than in the sepal (Figure 5K,L), and *SlMBP6* was highly expressed in petals and carpels (Figure 5M). In comparison, the other class E genes (*SlMADS1* and *SlMBP21*) were shown to exhibit higher expression levels in the sepal and carpel (Figure 5N,O).

In addition, the interaction networks of these 15 tomato MADS-box proteins were predicted by STRING software (Figure S4). The results showed that they established interactions with other proteins, directly or indirectly. The TAGL2, SlMBP3, TAGL1, and SlMBP22 proteins can interact directly with each other. Apart from that, the TAGL1 protein showed complex interactions with several other proteins, including the SlMBP3 and TAP3 proteins. The TM5 protein was also shown to directly interact with the TAP3 and TM6 proteins.

### 2.7. Differential Expression Analysis of Tomato MADS-Box Genes at Different Stages of Fruit Development and Ripening

**Figure 6:**
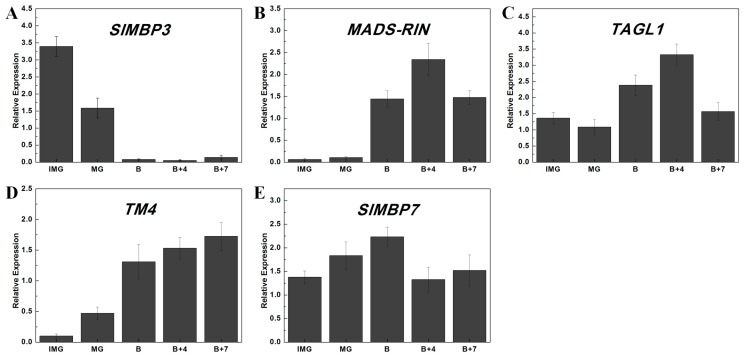
Relative expression of tomato MADS-box genes in difference stages of fruit development and ripening by qPCR analysis. Expression patterns of *SlMBP3* (**A**), *MADS-RIN* (**B**), *TAGL1* (**C**), *TM4* (**D**), and *SlMBP7*(**E**) in different organs, including mature green (MG), break (B), four days after break (B+4), and seven days after break (B+7). Each value represents the mean ± SE of three technical replicates of a single biological sample. The *SlCAC* gene of tomato was used as the internal standard.

According to the expression profiles predictions, we selected five tomato MADS-box genes (*SlMBP3*, *MADS-RIN*, *TAGL1*, *TM4*, and *SlMBP7*) that may take part in fruit development and ripening. We analyzed their expression patterns by qPCR at five different stages of fruit development and ripening, including the immature green (IMG), mature green (MG), and break (B) stages, as well as at four days after break (B+4) and seven days after break (B+7). *SlMBP3* exhibited a strikingly high expression level at the IMG stage, and showed extremely low expression at the other stages (Figure 6A). The expression levels of *MADS-RIN* and *TAGL1* exhibited an increasing tendency from the MG to the B+4 stage, and then decreased at B+7 (Figure 6B,C). *TM4* expression increased continuously during the process of fruit development and exhibited its maximum expression level at the B+7 stage (Figure 6D). Compared with the other three stages, the expression of *SlMBP7* was slightly higher at B (Figure 6E). These results indicated that the qPCR data were consistent with the predictions of expression profiles and that our predictions are suitable for investigating the expression patterns of tomato MADS-box genes.

## 3. Discussion

### 3.1. Characterization of MADS-Box Genes in Tomato

The MADS-box genes control diverse biological processes in plants, including vegetative growth and reproductive development. They mainly play key roles in the developmental processes of inflorescences, flowers, and fruits [78,79]. 24 MIKC^C^-type MADS-box transcription factors have been identified, and their functions and evolutions in tomatoes were thoroughly studied in 2006 [35]. However, the MIKC^C^-type MADS-box members are only part of the MADS-box transcription factor family, and to date, there has been no comparative report on the tomato MADS-box genes. It is well known that genome-wide analysis of gene families is a major and necessary approach to analyze the structure, evolution, and function of genes. In this study, 131 tomato MADS-box proteins were identified, and 96 new tomato MADS-box proteins with unknown functions were systemically named (Table 1). This study is the first comparative analysis of the tomato MADS-box gene family, and we believe that the resolving confusion in naming the genes will facilitate further functional analysis of the tomato MADS-box genes.

First, we presented the phylogenetic relationships of 131 tomato MADS-box proteins with Arabidopsis MADS-box proteins to classify the tomato MADS-box proteins into five subfamilies (MIKC, Mα, Mβ, Mγ, and Mδ), as shown Figure S1A. Compared with Arabidopsis, a larger number of MADS-box proteins were found in tomato. In total, 81 tomato MADS-box genes were determined to be type I genes, including the Mα, Mβ, Mγ, and Mδ groups, which is more than that in Arabidopsis. We speculate that tomato type I MADS-box genes may have a higher duplication rate and/or a lower gene loss rate after duplication. Nevertheless, 50 tomato MADS-box genes were classified as type II genes, including MIKC^c^ and MIKC*, which is comparable to that in Arabidopsis. These results indicate that the genes’ retention duplication have been different in various species, leading to different numbers of MAD-box genes among different species, with different evolutionary constraints [80]. Then, in order to investigate the phylogenetic relationships of MADS-box genes in tomato, a phylogenetic tree for two types of tomato MADS-box genes was constructed (Figure S1B,C). This showed that tomato MADS-box genes are conservative in subfamilies.

To obtain insight into the structural diversity of the tomato MADS-box genes, the intron–exon organization was analyzed (Figure 1). Previous studies have postulated that an intron-rich gene would lose multiple introns simultaneously by retrotransposition, thereby producing intron-less ancestral genes. In this study, we found that the Mα, Mβ and Mγ groups of the type I genes usually have no introns or one intron, which may experience the loss of multiple introns during MADS-box gene family diversification. In addition, the distribution of introns in tomato type I and type II genes were different, and the Mδ group of the type I and type II genes had more introns than the Mα, Mβ, and Mγ groups genes. Similar cases have also been detected in Arabidopsis and rice [7,13], suggesting the evolutionary conservation among plants. However, some close gene pairs showed different intron/exon arrangements, indicating that a more complicated gene structural evolution may exist in tomato MADS-box genes. The conserved motif analysis indicated that the same group contained most conserved motifs (Figure 2). The results suggested that these conserved motifs play important roles in group-specific functions. However, high structural divergence was found between the different groups. An analysis of the gene structures and conserved motifs could provide more clues about the evolutionary relationships of the MADS-box family in tomato.

Gene duplication (segmental duplication and tandem duplication) as well as transposition events were prevalent forces that result in the expansion of family members and genome complexity in eukaryotes [74]. The duplication of more than two genes located on one chromosome is confirmed as a tandem duplication event, whereas gene duplication that occurs on different chromosomes is identified as segmental duplication [73,81]. Both tandem and segmental duplication can play crucial roles in MADS-box gene expansion the tomato genome. In our study, a chromosomal location analysis of the tomato MADS-box genes showed that the MADS-box genes are distributed on 12 chromosomes (Figure 3). The tomato MADS-box genes had a high-density distribution on chromosome 1, which had 24 genes, suggesting that they might be caused by tandem duplications. The closely related tomato MADS-box genes formed tandem arrays on chromosomes 1, 3, 4, 10, and 12, which may help the tomato evolve distinct characterizations from other plants.

Since gene expression profiles can provide significant clues about gene function, the expression of tomato MADS-box genes in whole root (Rt), young leaf (YL), mature leaves (ML), young flower buds (YFB), fully open flowers (F), and five different fruit tissues were examined by transcription expression (RNA-seq) data. All the tested 124 tomato MAD-box genes that were expected to contain *SlMADS4*, *SlMADS11*, *SlMADS37*, *SlMADS44*, *SlMADS46*, *SlMADS56*, *SlMADS68*, *SlMADS70*, and *SlMADS89* showed distinct expression patterns (Figure 4). This finding may supply insight for future studies on the functions of MADS-box genes in tomato plant growth and development. For example, we found that the *SlMADS23* gene was only expressed in the root, so we speculate that the *SlMADS23* gene may play a key role in root growth and development. The roots of plants determined the capacity of plants to acquire and distribute nutrients and water, as well as provide a means to suit the environmental conditions. Thus, the root architecture is extremely important for plant development and breeding. In the future, we will verify whether *SlMADS23* is related to root growth by constructing a *SlMADS23* overexpression vector and generating transgenic overexpression tomato plants to study the regulation of *SlMADS23* gene on root growth and development. In addition, performing *SlMADS23* gene mutagenesis with the CRISPR/Cas9 system transformation method may also prove to be a helpful strategy.

### 3.2. Prediction of MADS-Box Genes Involved in the Regulation of Flower Development and Floral Organ Identity

An investigation of the genetic and molecular basis of flower development and floral organ identity in Arabidopsis and petunia suggested that MADS-box genes play fundamental roles in floral organ identity and flower development [82]. It has been confirmed that five classes of MADS-box genes (A–E) were involved in specifying floral organ identity [83,84,85]. In Arabidopsis, the class A genes (*AP1* and *AP2*), the class B genes (*AP3* and *PI*), the class C gene (*AG*), the class D gene (*AGL11*), and the class E gene (*SEP1*, *SEP2*, *SEP3,* and *SEP4*) were MADS-box genes, which have been reported to be involved in the regulation of floral organ development [83,86]. In petunia, lots of MADS-box genes, including the class A genes PETUNIA FLOWERING GENE (*PFG*), FLORAL BINDING PROTEIN 26 (*FBP26*), and *FBP29*, the class B genes *TM6*, *PMADS1*/*GP*, *PMADS2,* and *FBP1*, the class C genes *PMADS3*, *FBP6,* and *FBP24*, the class D genes *FBP11* and *FBP7,* as well as *FBP2*, *FBP4*, *FBP5*, *FBP9*, and the class E genes *FBP23*, *PMADS4,* and *PMADS12* played important roles in flower development [17,87].

In this paper, we investigated the tomato MADS-box genes’ phylogenetic relationships with the petunia hybrid to select 15 tomato MADS-box genes that may play specific roles in flower development (Table S2). According to the expression profile predictions shown in Figure 4, the highest expression values for most of the genes (*MADS-MC TAP3*, *TM6*, *SlMBP1*, *SlMBP2*, *TAG1*, *SlMBP22*, *SlMBP21,* and *SlMBP6*) were observed in flower development stages. Furthermore, qPCR was used to study the expression patterns of four whorls of floral organs (sepal, petal, stamen, and carpel) in these 15 tomato MADS-box genes.

*AP1* is an Arabidopsis A class gene, which conferred sepal identity in the first floral [88]. In petunia, the three genes *PFG*, *FBP26,* and *FBP29* have been identified, which were orthologs of *AP1*/*SQUA* in Arabidopsis [17]. Our phylogenetic analysis showed that *MADS-MC* and *SlMBP20* belonged to this clade (Figure S3) and their expression were particularly high in sepal, suggesting that they might play a similar role to *AP1* (Figure 5A,B). The class B genes were involved in the identification of petal and stamen in angiosperms [89]. Regarding the class B genes, five close homologs of petunia—TOMATO MADS-BOX GENE6 (*TM6)*, GREEN PETAL (*GP*)/ PETUNIA MADS BOX GENE 1 (*PMADS1*), *PMADS2,* and *FBP1*—were found in tomato [77]. The qPCR analysis showed that *TAP3*, *SlMBP2,* and *SlMBP1* have petal and stamen specific expression, while the *TM6*/*TDR6* transcripts were mainly detected in the petal and carpel (Figure 5C–F). These results were similar to the homologous genes of that in petunia [77]. Two tomato MADS-box genes *TAG1* and *TAGL1*, which were involved in C functions, were from the monophyletic *AGAMOUS* (*AG*) subfamily. These two genes were mainly expressed in stamens and carpels (Figure 5G,H), which is consistent with their function in specifying stamen and carpel development [52]. *SlMBP3* and *SlMBP22*, which are highly homologous to two petunia class D MADS-box genes, *FBP11* and *FBP7*, were shown to be separately and exclusively expressed in carpel (Figure 5L,I). The result suggested that *SlMBP3* and *SlMBP22* may have similar functions to the petunia *FBP7* and *FBP11* genes, which are related to the establishment of real ovules or carpel-like structures [90]. Arabidopsis *SEP* genes, the typical class E genes, were necessary for the specification of sepal, petal, stamen, and carpel identity with interaction with the class A, B, C, and D genes [16]. In petunia, seven class E genes (*FBP2*, *FBP4*, *FBP23*, *FBP5*, *FBP9*, *pMADS12,* and *pMADS4*) have been determined that belong to the *SEPALLATA* (*SEP*) clade [32]. The tomato *TAGL2, TM5*, *SlMADS1*, *SlMBP21,* and *SlMBP6* genes were homologous to these petunia class E genes (Figure S3), and some differences in expression patterns have been observed (Figure 5J–N), indicating that these five tomato class E genes may be involved in multiple floral organ identity. Thus, we believe that the expression patterns of the tomato MADS-box genes identified in our study will be an important tool for understanding the flower development mechanisms in tomato. Previous reports have found that the MADS-box proteins are able to form multiple homologous or heterodimeric complexes in plants, and the combinatorial MADS-box proteins are often deriving their regulatory specificity from other DNA binding or accessory factors. To understand how the tomato MADS-box genes can act in flower development and floral organ identity, it is necessary to identify the network of protein–protein interactions amongst them. Therefore, the predicted interaction networks of the 15 tomato MADS-box proteins, which are involved in floral organ development, were analyzed in our report (Figure S4). In many domesticated crops, it’s an important way to select inflorescence architecture with improved flower production and yield. In tomato, SlMBP21 forms protein complexes with JOINTLESS and MACROCALYX as a transcription activator for tomato flower abscission zone development [50], because SlMBP21, J, and MC have a common function in the development of the tomato flower abscission zone. In breeding, altering any of these genes will have the function on plant growth. In this study, the predicted interaction networks may help us to understand how the tomato MADS-box genes can act in flower development and floral organ identity.

### 3.3. Potential Functions of Tomato MADS-Box Genes during Fruit Development

To better understand the roles of tomato MADS-box genes in fruit development and ripening, we selected five tomato MADS-box genes (*SlMBP3*, *MADS-RIN*, *TAGL1*, *TM4*, and *SlMBP7*) that were predicted to abundantly expressed at different stages of fruit development and ripening. Then, we detected their relative expression level in fruits samples from five different stages of development by qPCR (Figure 6). As shown in Figure 6A, the expression level of *SlMBP3* was found to be higher at the MG stage than at the other stages. We found that the expression levels of *MADS-RIN* and *TAGL1* exhibited an increasing tendency during the transition from the MG stage to the B+4 stage, and then decreased at the B+7 stage (Figure 6B,C). Moreover, the expression level of *TM4* was found to increase continuously in the process of fruit development (Figure 6D). The *SlMBP7* gene showed a relatively high expression level at the B stage (Figure 6E). Recent reports have identified a number of MADS-box genes that are required for the regulation of fruit development and ripening. One of the most representative is the tomato *MADS-RIN* gene, which is one of the earliest acting ripening regulators, and plays crucial roles in fruit ripening through ethylene dependent and independent ripening regulatory pathways [19,91]. In addition, *TAGL1*, *TM4,* and *SlMBP7* have been found to regulate fruit ripening in tomato [21,22]. Since our results were consistent with the functional research of these genes, the *SlMBP3* gene was predicted to be particularly high in the MG stage, indicating that it might play an important function in fruit development and ripening. These results help to advance our understanding of the function of MADS-box genes in the regulation of fruit developmental and ripening processes in tomato.

## 4. Materials and Methods

### 4.1. Plant Material and Growth Condition

In this article, tomatoes (*Solanum lycopersicum*, ‘Ailsa Craig’ AC^++^) from Laboratory of molecular biology of tomato, Bioengineering College, Chongqing University, Chongqing, China, were grown in controlled greenhouse conditions of a 16-h day (25 °C)/8-h night (18 °C) cycle, 80% humidity, a 250-μmol·m^−2^·s^−1^ light intensity, and were managed routinely. The tomato flowers were tagged at anthesis and floral organs: sepals (Se), petals (Pe), stamens (St), and carpels (Ca) were collected from flowers at anthesis. The fruit color and days post-anthesis (DPA) were used to differentiate the ripening days of tomato fruits. We defined 20 DPA as the immature green (IMG), and 35 DPA as the mature green (MG), at which point the fruits are green and shiny and no obvious color change is observed. The 38-DPA tomato fruits with color change from green to yellow were characterized as breaker (B) fruits. Besides, the samples of B+4 (4 days after breaker) fruits and B+7 (7 days after breaker) fruits were also used in our study. All the samples that we used were frozen in liquid nitrogen immediately and stored at –80 °C.

### 4.2. Identification of MADS-Box Genes in Tomato

The Sol Genomics Network (SGN, Available online: http://solgenomics.net/) and the National Center for Biotechnology Information (NCBI, Available online: https://www.ncbi.nlm.nih.gov/) database were used to comprehensively identify the whole MADS-box protein sequences of tomato. BLAST searches, using all the Arabidopsis and rice MADS-box protein sequences as queries, were performed to check the predicted tomato MADS-box protein sequences in the database. Subsequently, we further examined all the candidate protein sequences by the PROSITE (Available online: http://www.expasy.org/prosite/) and SMART (Available online: http://smart.embl-heidelberg.de/) databases for reliability. The tomato protein sequences, containing the typical conserved domain of the MADS-box protein family, were selected for amino acid sequence multiple alignment and phylogenetic tree analysis. Then, we obtained their DNA sequences according to their amino acid sequence from the SGN database. Additionally, the molecular weight and isoelectric points of tomato MADS-box proteins were detected by the ExPASy proteomics server.

### 4.3. Phylogenetic Analysis of Tomato MADS-Box Proteins

Multiple sequence alignment for the two groups of all the 131 tomato MADS-box genes (Table 1) was generated using ClustalX 1.81. The alignment results were used to conduct a phylogenetic tree by the MEGA5.02 program, and the evolutionary history was inferred using the neighbor-joining method. [92].

### 4.4. The Analysis of Gene Structure and Conserved Motif

The tomato MADS-box coding domain sequences (CDS) and corresponding genomic DNA sequences were collected from SGN and NCBI databases to predict gene structure. The online tool Gene Structure Display Server 2.0 (GSDS 2.0, Available online: http://gsds.cbi.pku.edu.cn/index.php), was used to construct an exon/intron map [93].

Conserved motifs of the tomato MADS-box protein sequences were identified by online software MEME Version 4.12.0 (Available online: http://meme-suite.org/tools/meme). It was performed with the following parameters: 10 different motifs, a motif width of 6–200 amino acids, and any number of repetitions. The SMART database was used to annotate the MEME motifs.

### 4.5. Chromosomal Locations and Identification of Interaction Network

To determine the chromosomal locations of tomato MADS-box genes, we obtained the physical genome annotation files from the SGN and NCBI database. The physical map of the tomato MADS-box genes was drawn by the Tomato-EXPEN 2000 (Available online: https://solgenomics.net/cview/map.pl).

The interaction network was conducted by STRING (functional protein association networks, Available online: https://string-db.org/) software using the search of multiple proteins sequences [94].

### 4.6. Digital Gene Expression Analysis of Tomato MADS-Box Genes

To obtain the expression profile of tomato MADS-box genes, the RNA-seq data based on the locus/gene names of SGN were analyzed. We downloaded the RNA-seq data from TFGD (the Tomato Functional Genomics Database), and the sequence data were obtained from various tissues in wild species LA1589 (*S. pimpinellifolium*). In addition, the data of the tomato lab and Tomato eFP Browser were also used to analyze the gene expression of tomato MADS-box genes. A heatmap was generated by Heml 1.0 (Heatmap illustrator, Available online: http://hemi.biocuckoo.org/) using the relative expression values or ratios of each tomato MADS-box gene [95].

### 4.7. Total RNA Extraction and qPCR Analysis

To study the expression patterns of the MADS-box genes involved in flower organ identity and fruit development in tomato, total RNA was extracted from the sepals (Se), petals (Pe), stamens (St), and carpels (Ca) of tomato and different developmental stages of tomato fruits at different developmental stages, including IMG (immature green), MG (mature green), B (breaker), B+4 (4 days after breaker), and B+7 (7 days after breaker) using RNAiso Plus (Takara) in accordance with the instructions. After DNase digestion (Promega, Madison, WI, USA), cDNA was synthesized with oligo(dT)20 as a primer for RNA reverse-transcription using M-MLV reverse transcriptase (Promega, Madison, WI, USA). For gene expression quantification, qPCR analysis was performed with the CFX96™ Real-Time System (Bio-Rad, Hercules, CA, USA) using the GoTaq qPCR Master Mix (Promega, Madison, WI, USA). First, 1.0 μL of mixture primers, 1.0 μL of cDNA, and 3.0 μL of ddH_2_O were used. NRT (no reverse transcription control) and NTC (no template control) experiments were performed to eliminate the genomic DNA and environment effects. The tomato *SlCAC* gene was used as an internal standard [96], and the 2^−ΔΔCT^ method was used to perform the relative gene expression levels analysis [97]. In addition, all the experiments were performed in three biological triplicates with three technical replicates. The standard curves were run at the same time. All the primers used were designed by Primer 5.0 software and are shown in Table S3.

### 4.8. Data Analysis

In this study, the mean values of data are presented as mean ± standard deviation. The Origin 8.0 software (Available online: https://www.originlab.com/) was used to perform the data analysis, and mean differences were significant by t-test (* *p* < 0.05).

## 5. Conclusions

In this study, a comprehensive and systematic analysis of the tomato *MADS-box* transcription factor family was first conducted. A total of 131 genes encoding MADS-box transcription factors, including 81 type I and 50 type II genes, were extensively identified in the tomato genome. Then, we classified the genes according to their phylogenetic relationships between tomato and Arabidopsis. The phylogenetic relationships, gene structures, conserved motifs, chromosomal distribution, and expression patterns of the genes were characterized. The 131 tomato MADS-box genes showed differential expression levels in different organs. Since the MADS-box genes are the most powerful TFs that regulate floral organ identity and fruit development and ripening in plants, we showed that 15 tomato MADS-box genes were involved in floral organ development, and we studied the expression of five tomato MADS-box genes in different stages of fruit development and ripening. These results provide evidence of the relationship between MADS-box genes and floral organ and fruit development. Our study provides comprehensive information on the tomato MADS-box gene family, enables a better understanding of the structure–function relationships among the tomato MADS-box gene family members, and lays a solid foundation of comprehensive functional characteristics in the tomato MADS-box gene family. Furthermore, our bioinformatics and evolutionary analysis will be helpful for better understanding the underlying evolutionary relationship of the MADS-box family in higher plants.

## Figures and Tables

**Table 1:** Overview of MADS-box genes identified in tomato. List of predicted genes and related information include gene name, gene locus, molecular details, classification of two types according to the phylogenetic analysis shown in Figure S1, homologs in Arabidopsis, as well as the reference of the gene function investigation. pI, isoelectric point; MW, molecular weight.

Gene Name	Gene Locus	Protein			Type	Reference
		Length (aa)	MW (Da)	pI		
*SlMBP1*/*SlGLO1*/*PI*/*LePI-B*	Solyc08g067230.2.1	210	24740.2	8.69	Type II	[36]
*SlMBP2*/*SlGLO2*/*LePI/TPI*	Solyc06g059970.2.1	214	24867.4	9.49	Type II	[37]
*SlMBP3/ SlAGL11*	Solyc06g064840.3.1	237	27469.6	9.25	Type II	[38]
*SlMBP6*/*SlAGL6*	Solyc01g093960.2.1	252	28608.5	8.37	Type II	[39,40,41]
*SlMBP7*/*LeFUL2*	Solyc03g114830.2.1	247	28614.3	9.31	Type II	[22,40,42,43,44]
*SlMBP8*	Solyc12g087830.1.1	198	22643.3	8.97	Type II	[45,46]
*SlMBP10*	Solyc02g065730.1.1	234	27368.1	10.12	Type II	
*SlMBP11/AGL15-like*	Solyc01g087990.2.1	271	30847	6.01	Type II	[47]
*SlMBP13*	Solyc08g080100.2.1	224	25839	9.76	Type II	
*SlMBP14*	*Solyc12g056460.1.1*	*206*	*23968.4*	*6.57*	Type II	
*SlMBP15*	Solyc12g087830.2.1	204	23667.2	8.35	Type II	
*SlMBP18*/*SlFYFL*	Solyc03g006830.2.1	222	25369.2	9.46	Type II	[48]
*SlMBP19*	Solyc06g035570.1.1	54	6224.26	11.29	Type I	
*SlMBP20*	Solyc02g089210.2.1	250	28589.2	9.87	Type II	[44]
*SlMBP21*	Solyc12g038510.1.1	250	28442.4	9.26	Type II	[49,50,51]
*SlMBP22*	Solyc11g005120.1.1	238	27791.6	6.96	Type II	
*SlMBP23*/*TDR3*	Solyc10g017630.2.1	166	19196.3	9.49	Type II	
*SlMBP24*	Solyc04g076280.3.1	235	26483.6	7.67	Type I	
*SlMBP25*	Solyc05g015730.1.1	80	9311.97	11.01	Type II	
*TAG1*	Solyc02g071730.2.1	248	28723.6	9.97	Type II	[52,53]
*TAGL1*	Solyc07g055920.2.1	267	29940.5	9.56	Type II	[20,21,52,54]
*TAGL2*	Solyc05g015750.2.1	241	27579.3	9.07	Type II	[55,56]
*TAGL11*	Solyc11g028020.1.1	223	26051.7	9.76	Type II	[38,56]
*TAGL12*	Solyc11g032100.1.1	201	23104.8	6.94	Type II	[56]
*TAP3*/*LeAP3*/*LeDEF*	Solyc04g081000.2.1	228	26478.2	9.76	Type II	[54,57,58,59]
*MADS-RIN*	Solyc05g012020.2.1	242	27968.4	8.00	Type II	[19,60,61,62,63]
*MADS-MC*	Solyc05g056620.1.1	244	28660.6	8.44	Type II	[64]
*JOINTLESS*	Solyc11g010570.1.1	265	30426.3	7.39	Type II	[64,65,66,67,68]
*LeAP1*	Solyc05g012020.3.1	213	25077.3	5.47	Type II	[40]
*TM4*/*TDR4*/*LeFUL1*	Solyc06g069430.2.1	245	28290	9.57	Type II	[22,42,43,59]
*TM5*/*TDR5*/*LeSEP3*	Solyc05g015750.3.1	224	25999.3	9.79	Type II	[40]
*TM6*/*TDR6*	Solyc02g084630.2.1	225	26092.7	9.71	Type II	[69]
*TM8*/*TDR8*	Solyc03g019710.2.1	177	20703.9	10.44	Type II	[70]
*SlMADS1*	Solyc03g114840.2.1	246	28398.2	8.88	Type II	[55,71]
*SlMADS2*	Solyc01g060300.1.1	211	23122.6	9.36	Type I	
*SlMADS3*	Solyc01g060310.1.1	139	15117.5	8.99	Type I	
*SlMADS4*	Solyc01g060284.1.1	197	21565.5	8.63	Type I	
*SlMADS5*	Solyc01g066500.1.1	309	34544.4	4.75	Type I	
*SlMADS6*/*TM29*/*LeSEP1*	Solyc02g089200.2.1	246	28481.3	8.29	Type II	[39,41]
*SlMADS7*	Solyc01g103870.1.1	125	14435.6	9.54	Type I	
*SlMADS8*	Solyc06g071300.1.1	130	15009.1	5.14	Type I	
*SlMADS9*	Solyc03g007020.1.1	215	24702	9.21	Type I	
*SlMADS10*	Solyc04g064860.1.1	233	25871.3	7.92	Type I	
*SlMADS11*	Solyc04g054517.1.1	138	15663.1	7.75	Type I	
*SlMADS12*	Solyc04g056550.1.1	182	20838.7	5.14	Type I	
*SlMADS13*	Solyc04g056740.1.1	180	20521.5	7.44	Type I	
*SlMADS14*	Solyc06g054680.1.1	180	21152.8	7.19	Type I	
*SlMADS15*	Solyc06g059780.1.1	180	21057.6	6.37	Type I	
*SlMADS16*	Solyc10g050950.1.1	161	18602.2	6.80	Type I	
*SlMADS17*	Solyc10g050900.1.1	169	19796.5	7.83	Type I	
*SlMADS18*	Solyc10g050940.1.1	169	19623.3	7.77	Type I	
*SlMADS19*	Solyc01g097850.1.1	172	20073.9	7.35	Type I	
*SlMADS20*	Solyc03g034260.1.1	184	21441	6.29	Type I	
*SlMADS21*	Solyc10g018070.1.1	119	13063.9	10.14	Type I	
*SlMADS22*	Solyc10g018080.1.1	151	17218.5	7.04	Type I	
*SlMADS23*	Solyc10g018110.1.1	184	20762.4	6.55	Type I	
*SlMADS24*	Solyc04g025970.1.1	117	13333.4	10.16	Type I	
*SlMADS25*	Solyc01g010300.1.1	88	10052.7	10.79	Type I	
*SlMADS26*	Solyc02g032000.1.1	97	10944.7	10.32	Type I	
*SlMADS27*	Solyc03g119680.1.1	177	20293.2	7.26	Type I	
*SlMADS28*	Solyc09g061950.1.1	173	20138.1	9.33	Type I	
*SlMADS29*	Solyc01g106710.1.1	221	25105.7	10.23	Type I	
*SlMADS30*	Solyc04g025030.1.1	135	15411	10.37	Type I	
*SlMADS31*	Solyc04g025110.1.1	130	14313.7	10.26	Type I	
*SlMADS32*	Solyc01g106720.1.1	211	23531.2	10.35	Type I	
*SlMADS33*	Solyc01g106730.1.1	221	23888.6	10.14	Type I	
*SlMADS34*	Solyc04g047870.1.1	190	21695.2	10.70	Type I	
*SlMADS35*	Solyc10g012180.1.1	167	18824.5	5.38	Type I	
*SlMADS36*	Solyc10g012200.1.1	167	19113.1	8.98	Type I	
*SlMADS37*	Solyc11g067163.1.1	166	18919.9	7.67	Type I	
*SlMADS38*	Solyc01g066730.2.1	300	33023.4	5.04	Type I	
*SlMADS39*	Solyc01g098070.1.1	155	17200.8	10.09	Type I	
*SlMADS40*	Solyc01g098060.1.1	152	17191.5	9.84	Type I	
*SlMADS41*	Solyc03g062820.1.1	145	16350.8	9.23	Type I	
*SlMADS42*	Solyc01g098050.1.1	160	18178	10.45	Type I	
*SlMADS43*	Solyc05g013370.1.1	232	26476.7	5.46	Type I	
*SlMADS44*	Solyc05g046345.1.1	250	28489.8	5.47	Type I	
*SlMADS45*	Solyc04g025050.1.1	56	6259.52	11.07	Type I	
*SlMADS46*	Solyc07g017343.1.1	226	26532.7	5.2	Type I	
*SlMADS47*	Solyc00g179240.1.1	55	6310.11	11.16	Type I	
*SlMADS48*	Solyc07g052707.1.1	195	21878.8	4.86	Type I	
*SlMADS49*	Solyc07g052700.2.1	190	21334.2	7.41	Type I	
*SlMADS50*	Solyc11g069770.2.1	175	20171.9	5.45	Type I	
*SlMADS51*	Solyc01g106700.2.1	213	24279.5	7.35	Type I	
*SlMADS52*	Solyc03g115910.1.1	417	47275.1	5.82	Type I	
*SlMADS53*	Solyc10g012380.1.1	149	17086.7	5.29	Type I	
*SlMADS54*	Solyc10g012390.1.1	149	17086.7	5.29	Type I	
*SlMADS55*	Solyc11g069770.1.1	181	20857.8	5.20	Type I	
*SlMADS56*	Solyc12g042967.1.1	225	25552.3	9.05	Type I	
*SlMADS57*	Solyc11g020620.1.1	342	38747.9	5.42	Type I	
*SlMADS58*	Solyc11g020660.1.1	312	35558.7	4.61	Type I	
*SlMADS59*	Solyc11g020320.1.1	275	31757.7	6.68	Type I	
*SlMADS60*	Solyc01g103550.1.1	311	35839.7	7.87	Type I	
*SlMADS61*	Solyc01g060310.2.1	136	15230.6	6.16	Type I	
*SlMADS62*	Solyc12g016170.1.1	219	25087.7	6.57	Type I	
*SlMADS63*	Solyc12g016180.1.1	219	25207.8	6.57	Type I	
*SlMADS64*	Solyc12g016150.1.1	154	17645.1	5.38	Type I	
*SlMADS65*	Solyc12g017300.1.1	154	17740.1	5.11	Type I	
*SlMADS66*	Solyc12g005210.1.1	88	9985.65	10.41	Type I	
*SlMADS67*	Solyc01g102260.2.1	249	27882.8	9.66	Type I	
*SlMADS68*	Solyc05g047712.1.1	156	18199.1	8.89	Type I	
*SlMADS69*	Solyc07g043080.1.1	215	24384.8	6.86	Type I	
*SlMADS70*	Solyc06g034317.1.1	153	16949.3	4.41	Type I	
*SlMADS71*	Solyc06g048380.1.1	176	20038.6	4.90	Type I	
*SlMADS72*	Solyc06g048380.2.1	132	15027	4.82	Type I	
*SlMADS73*	Solyc06g033820.1.1	196	22657.4	7.25	Type I	
*SlMADS74*	Solyc06g033830.1.1	113	13058.8	8.44	Type I	
*SlMADS75*	Solyc07g052700.3.1	197	22133.2	8.44	Type I	
*SlMADS76*	Solyc03g115910.2.1	434	49552.6	5.6	Type I	
*SlMADS77*	Solyc12g087820.1.1	106	11969.2	11.03	Type I	
*SlMADS78*	Solyc05g051830.2.1	389	44560	5.91	Type I	
*SlMADS79*	Solyc10g017640.1.1	61	7032.22	11.24	Type I	
*SlMADS80*	Solyc04g076680.2.1	63	70492.3	11.03	Type I	
*SlMADS81*	Solyc12g088080.1.1	97	11295.2	10.59	Type I	
*SlMADS82*	Solyc01g105800.2.1	279	32104.7	9.11	Type II	
*SlMADS83*	Solyc01g106170.2.1	148	17032.2	10.98	Type II	
*SlMADS84*	Solyc10g080030.1.1	229	25791.3	6.37	Type II	
*SlMADS85*	Solyc01g105810.3.1	215	24518.2	9.22	Type II	
*SlMADS86*	Solyc04g078300.3.1	145	16547.2	9.9	Type II	
*SlMADS87*	Solyc04g078300.2.1	334	38130.7	5.96	Type II	
*SlMADS88*	Solyc03g006830.3.1	209	24310.2	9.43	Type II	
*SlMADS89*	Solyc10g044967.1.1	226	26172	6.14	Type II	
*SlMADS90*	Solyc11g032100.2.1	201	23104.8	6.45	Type II	
*SlMADS91*	Solyc05g015720.2.1	204	23433.5	5.36	Type II	
*SlMADS92*	Solyc01g093960.3.1	242	27359.1	8.19	Type II	
*SlMADS93*	Solyc02g091550.2.1	249	28342.2	9.07	Type II	
*SlMADS94*	Solyc02g091550.1.1	235	27198.1	9.65	Type II	
*SlMADS95*	Solyc04g076700.3.1	226	25933.3	8.59	Type II	
*SlMADS96*	Solyc03g019720.3.1	193	22631.1	9.88	Type II	
*SlMADS97*	Solyc12g088090.2.1	239	27695.8	9.84	Type II	
*SlMADS98/SlCMB1*	Solyc04g005320.2.1	238	27522.1	8.60	Type II	[72]

## Supplementary Materials

Supplementary materials can be found at https://www.mdpi.com/1422-0067/20/12/2961/s1.
