# Supplementary material for: Genome-Wide Analysis of the MADS-Box Transcription Factor Family in Solanum lycopersicum
Source: Int J Mol Sci. 2019 Jun 18;20(12):2961. doi: 10.3390/ijms20122961 (PMC6627509; doi:10.3390/ijms20122961)
Supplement: Supplementary file 1 [file ijms-20-02961-s001.pdf]

Supplementary Table 1. Homologous genes of tomato MADS-box family genes in Arabidopsis and the references for the study of the functions in homologous genes.

Gene Name	Homologous Gene	Reference
SlMBP1/SlGLO1/PI/LePI-B	***PISTILLATA***	[1,2]
SlMBP2/SlGLO2/LePI/TPI	*PISTILLATA*	[1,2]
SlMBP3/ SlAGL11	*AGL11*	[3-5]
SlMBP6/SlAGL6	AGL6	[6-8]
SlMBP7/LeFUL2	PISTILLATA	[9]
SlMBP8	AGL70	[10]
SlMBP10	AGL8	[11]
SlMBP11/ AGL15-like	AGL15	[12,13]
SlMBP13	-	-
SlMBP14	AGL20	*[14]*
SlMBP15	AGL70	[10]
SlMBP18/SlFYFL	AGL42	[15]
SlMBP19	AGL42	[15]
SlMBP20	AGL7/AP1	[16-19]
SlMBP21	AGL3	[20]
SlMBP22	AGL32/TT16	[21,22]
SlMBP23/TDR3	-	-
SlMBP24	AGL22/SVP	[23-26]
SlMBP25	-	-
TAG1	AGAMOUS	[27-30]
TAGL1	AGL1	[31]
TAGL2	AGL9	[32]
TAGL11	AGL11	[3-5]
TAGL12	AGL12	[33]
TAP3/LeAP3/LeDEF	AP3	[34]
MADS-RIN	AGL2	[35]
MADS-MC	AGL79	
JOINTLESS	AGL22/SVP	[23-26]
LeAP1	AGL10/CAL	[36,37]
TM4/TDR4/LeFUL1	AGL79	[38]
TM5/TDR5/LeSEP3	AGL9	[32]
TM6/TDR6	AGL32/TT16	[21,22]
TM8/TDR8	-	-
SlMADS1	AGL3	[20]
SlMADS2	AGL62	[39]
SlMADS3	AGL62	[39]
SlMADS4	AGL62	[39]
SlMADS5	AGL40	-
SlMADS6/ TM29/LeSEP1	AGL4	45
SlMADS7	AGL98	-
SlMADS8	AGL98	-
SlMADS9	AGL61	46
SlMADS10	AGL62	[39]
SlMADS11	AGL104	-

Gene Name	Homologous Gene	Reference
SlMADS12	AGL57	-
SlMADS13	AGL62	[39]
SlMADS14	AGL62	[39]
SlMADS15	AGL61	-
SlMADS16	AGL57	-
SlMADS17	AGL57	-
SlMADS18	AGL62	[39]
SlMADS19	AGL62	[39]
SlMADS20	AGL61	-
SlMADS21	AGL57	-
SlMADS22	AGL29	-
SlMADS23	AGL29	-
SlMADS24	AGL62	[39]
SlMADS25	AGL20	*14*
SlMADS26	AGL40	-
SlMADS27	AGL61	-
SlMADS28	AGL29	-
SlMADS29	AGL62	[39]
SlMADS30	AGL62	[39]
SlMADS31	AGL62	[39]
SlMADS32	AGL62	[39]
SlMADS33	AGL62	[39]
SlMADS34	AGL62	[39]
SlMADS35	AGL50	-
SlMADS36	AGL49	-
SlMADS37	AGL45	-
SlMADS38	AGL60	-
SlMADS39	AGL60	-
SlMADS40	AGL60	-
SlMADS41	AGL62	[39]
SlMADS42	AGL23	[40]
SlMADS43	AGL103	-
SlMADS44	AGL80	[41,42]
SlMADS45	AGL62	[39]
SlMADS46	AGL79	-
SlMADS47	AGL14	[43,44]
SlMADS48	AGL66	-
SlMADS49	AGL66	-
SlMADS50	AGL62	[39]
SlMADS51	AGL62	[39]
SlMADS52	AGL65	-
SlMADS53	-	-
SlMADS54	-	-
SlMADS55	AGL62	[39]
SlMADS56	AGL80	[41,42]
SlMADS57	AGL47	-

Gene Name	Homologous Gene	Reference
SlMADS58	AGL92	-
SlMADS59	AGL92	-
SlMADS60	AGL92	-
SlMADS61	AGL61	-
SlMADS62	AGL86	-
SlMADS63	AGL48	-
SlMADS64	AGL96	-
SlMADS65	AGL96	-
SlMADS66	AGL46	-
SlMADS67	AGL80	[41,42]
SlMADS68	AGL38	-
SlMADS69	AGL80	[41,42]
SlMADS70	AGL79	[38]
SlMADS71	AGL66	-
SlMADS72	AGL94	-
SlMADS73	AGL24	[45,46]
SlMADS74	AGL79	[38]
SlMADS75	AGL66	-
SlMADS76	AGL94	-
SlMADS77	-	-
SlMADS78	AGL104	-
SlMADS79	AGL19	[47-49]
SlMADS80	AGL16	[49]
SlMADS81	AGL44	[50]
SlMADS82	AGL22/SVP	[23]
SlMADS83	AGL44	[50]
SlMADS84	AGL18	60-82
SlMADS85	AGL22/SVP	[23-26]
SlMADS86	AGL22/SVP	[23-26]
SlMADS87	AGL104	-
SlMADS88	AGL42	[15]
SlMADS89	AGL21	[51,52]
SlMADS90	AGL12	[33]
SlMADS91	AGL31	[53,54]
SlMADS92	AGL6	[6-8]
SlMADS93	AGL44	[50]
SlMADS94	AGL44	[50]
SlMADS95	AGL44	[50]
SlMADS96	AGL22/SVP	[23-26]
SlMADS97	AGL16	[54]
SlMADS98/SlCMB1	AGL22/SVP	[23-26]

References

1. Jack, T.; Brockman, L.L.; Meyerowitz, E.M. The homeotic gene APETALA3 of Arabidopsis thaliana encodes a MADS box and is expressed in petals and stamens. *Cell* **1992**, *68*, 683-697.
2. Yang, Y.; Fanning, L.; Jack, T. The K domain mediates heterodimerization of the Arabidopsis floral organ identity proteins, APETALA3 and PISTILLATA. *Plant Journal* **2003**, *33*, 47-59.

3. Ezquer, I.; Mizzotti, C.; Nguema-Ona, E.; Gott, M.; Beauzamy, L.n.; Viana, V.E.; Dubrulle, N.; Oliveira, A.C.D.; Caporali, E.; Koroney, A.S. The Developmental Regulator SEEDSTICK Controls Structural and Mechanical Properties of the Arabidopsis Seed Coat. *Plant Cell* **2016**, *28*, 2478.
4. Mizzotti, C.; Ezquer, I.; Paolo, D.; Ruedaromero, P.; Guerra, R.F.; Battaglia, R.; Rogachev, I.; Aharoni, A.; Kater, M.M.; Caporali, E. SEEDSTICK is a Master Regulator of Development and Metabolism in the Arabidopsis Seed Coat. *Plos Genetics* **2014**, *10*, e1004856.
5. Mizzotti, C.; Mendes, M.A.; Caporali, E.; Schnittger, A.; Kater, M.M.; Battaglia, R.; Colombo, L. The MADS box genes SEEDSTICK and ARABIDOPSIS Bsister play a maternal role in fertilization and seed development. *Plant Journal* **2012**, *70*, 409-420.
6. Huang, X.; Effgen, S.; Meyer, R.C.; Theres, K.; Koornneef, M. Epistatic natural allelic variation reveals a function of AGAMOUS-LIKE6 in axillary bud formation in Arabidopsis. *Plant Cell* **2012**, *24*, 2364-2379.
7. Yoo, S.K.; Hong, S.M.; Lee, J.S.; Ji, H.A. A genetic screen for leaf movement mutants identifies a potential role for AGAMOUS-LIKE 6 (AGL 6) in circadian-clock control. *Molecules & Cells* **2011**, *31*, 281-287.
8. Koo, S.C.; Bracko, O.; Park, M.S.; Schwab, R.; Chun, H.J.; Park, K.M.; Seo, J.S.; Grbic, V.; Balasubramanian, S.; Schmid, M. Control of lateral organ development and flowering time by the Arabidopsis thaliana MADS-box gene AGAMOUS-LIKE6. *Plant Journal for Cell & Molecular Biology* **2010**, *62*, 807-816.
9. Goto, K.; Meyerowitz, E.M. Function and regulation of the Arabidopsis floral homeotic gene PISTILLATA. *Genes & Development* **1994**, *8*, 1548.
10. Ratcliffe, O.J.; Kumimoto, R.W.; Wong, B.J.; Riechmann, J.L. Analysis of the Arabidopsis MADS AFFECTING FLOWERING gene family: MAF2 prevents vernalization by short periods of cold. *Plant Cell* **2003**, *15*, 1159-1169.
11. Mandel, M.A.; Yanofsky, M.F. The Arabidopsis AGL8 MADS box gene is expressed in inflorescence meristems and is negatively regulated by APETALA1. *Plant Cell* **1995**, *7*, 1763-1771.
12. Adamczyk, B.J.; Al, E. The MADS domain factors AGL15 and AGL18 act redundantly as repressors of the floral transition in Arabidopsis. *Plant Journal for Cell & Molecular Biology* **2010**, *50*, 1007-1019.
13. Heck, G.R.; Perry, S.E.; Nichols, K.W.; Fernandez, D.E. AGL15, a MADS domain protein expressed in developing embryos. *Plant Cell* **1995**, *7*, 1271-1282.
14. Kimura, Y.; Aoki, S.; Ando, E.; Kitatsuji, A.; Watanabe, A.; Ohnishi, M.; Takahashi, K.; Inoue, S.; Nakamichi, N.; Tamada, Y. A Flowering Integrator, SOC1, Affects Stomatal Opening in Arabidopsis thaliana. *Plant & Cell Physiology* **2015**, *56*, 640-649.
15. Dorcafornell, C.; Gregis, V.; Grandi, V.; Coupland, G.; Colombo, L.; Kater, M.M. The Arabidopsis SOC1-like genes AGL42, AGL71 and AGL72 promote flowering in the shoot apical and axillary meristems. *Plant Journal* **2011**, *67*, 1006-1017.
16. Han, Y.; Jiao, Y. APETALA1 establishes determinate floral meristem through regulating cytokinins homeostasis in Arabidopsis. *Plant Signal Behav* **2015**, *10*, .
17. Han, Y.; Zhang, C.; Yang, H.; Jiao, Y. Cytokinin pathway mediates APETALA1 function in the establishment of determinate floral meristems in Arabidopsis. *Proceedings of the National Academy of Sciences of the United States of America* **2014**, *111*, 6840.
18. UN, K.o.; Kupriianova, E.V.; Ezhova, T.A. [Different action of the APETALA1 gene on the development of reproductive organs in flowers of the abruptus mutant of Arabidopsis thaliana (L.) Heynh]. *Ontogenez* **2011**, *42*, 307.
19. Kaufmann, K.; Wellmer, F.; Mu, J.M.; Ferrier, T.; Wuest, S.E.; Kumar, V.; Serranomislata, A.; Madue, F.; Krajewski, P.; Meyerowitz, E.M. Orchestration of floral initiation by APETALA1. *Science* **2010**, *328*, 85-89.
20. Ditta, G.; Pinyopich, A.; Robles, P.; Pelaz, S.; Yanofsky, M.F. The SEP4 gene of Arabidopsis thaliana functions in floral organ and meristem identity. *Current Biology* **2004**, *14*, 1935-1940.
21. Nesi, N.; Debeaujon, I.; Jond, C.; Stewart, A.J.; Jenkins, G.I.; Caboche, M.; Lepiniec, L.c. The TRANSPARENT TESTA16 locus encodes the ARABIDOPSIS BSISTER MADS domain protein and is required for proper development and pigmentation of the seed coat. *Plant Cell* **2002**, *14*, 2463-2479.
22. Xu, W.; Bobet, S.; Gourrierec, J.L.; Grain, D.; Vos, D.l.D.; Berger, A.; Salsac, F.; Kelemen, Z.; Boucherez, J.; Rolland, A.l. TRANSPARENT TESTA 16 and 15 act through different mechanisms to control proanthocyanidin accumulation in Arabidopsis testa. *Journal of Experimental Botany* **2017**, *68*, 2859-2870.
23. Wilson, D.C.; Kempthorne, C.J.; Carella, P.; Liscombe, D.K.; Cameron, R. Age-Related Resistance in Arabidopsis thaliana Involves the MADS-domain Transcription Factor SHORT VEGETATIVE PHASE and Direct Action of Salicylic Acid on Pseudomonas syringae. *Mol Plant Microbe Interact* **2017**,

MPMI-07-17-0172-R.

24. Bechtold, U.; Penfold, C.A.; Jenkins, D.J.; Legaie, R.; Moore, J.D.; Lawson, T.; Matthews, J.S.; Vialet-Chabrand, S.R.; Baxter, L.; Subramaniam, S. Time-series transcriptomics reveals that AGAMOUS-LIKE22 links primary metabolism to developmental processes in drought-stressed Arabidopsis. *Plant Cell* **2016**, *28*, 345.
25. Gregis, V.; Andrs, F.; Sessa, A.; Guerra, R.F.; Simonini, S.; Mateos, J.L.; Torti, S.; Zambelli, F.; Prazzoli, G.M.; Bjerkan, K.N. Identification of pathways directly regulated by SHORT VEGETATIVE PHASE during vegetative and reproductive development in Arabidopsis. *Genome Biology* **2013**, *14*, R56-R56.
26. Cho, H.J.; Kim, J.J.; Lee, J.H.; Kim, W.; Jung, J.H.; Park, C.M.; Ji, H.A. SHORT VEGETATIVE PHASE (SVP) protein negatively regulates miR172 transcription via direct binding to the priΓÇÉmiR172a promoter in Arabidopsis. *Febs Letters* **2012**, *586*, 2332-2337.
27. Uemura, A.; Yamaguchi, N.; Xu, Y.; Wee, W.; Ichihashi, Y.; Suzuki, T.; Shibata, A.; Shirasu, K.; Ito, T. Regulation of floral meristem activity through the interaction of AGAMOUS, SUPERMAN, and CLAVATA3 in Arabidopsis. *Plant Reproduction* **2018**, *31*, 89-105.
28. Cartolano, M.; Efremova, N.; Kuckenberg, M.; Raman, S.; Schwarzsommer, Z. Enhanced AGAMOUS expression in the centre of the Arabidopsis flower causes ectopic expression over its outer expression boundaries. *Planta* **2009**, *230*, 857-862.
29. Ito, T.; Wellmer, F.; Yu, H.; Das, P.; Ito, N.; Alvesferreira, M.; Riechmann, J.L.; Meyerowitz, E.M. The homeotic protein AGAMOUS controls microsporogenesis by regulation of SPOROCYTELESS. *Nature* **2004**, *430*, 356-360.
30. G ├ │ mezmena, C.n.; Folter, S.D.; Costa, M.M.R.; Angenent, G.C.; Sablowski, R. Transcriptional program controlled by the floral homeotic gene AGAMOUS during early organogenesis. *Development* **2005**, *132*, 429-438.
31. Colombo, M.; Brambilla, V.; Marcheselli, R.; Caporali, E.; Kater, M.M.; Colombo, L. A new role for the SHATTERPROOF genes during Arabidopsis gynoecium development. *Developmental Biology* **2010**, *337*, 294-302.
32. Lee, J.H.; Kim, J.J.; Ji, H.A. Role of SEPALLATA3 (SEP3) as a downstream gene of miR156-SPL3-FT circuitry in ambient temperature-responsive flowering. *Plant Signaling & Behavior* **2012**, *7*, 1151-1154.
33. Tapia-L ├ú┬ │ Pez, R.; Garc ├ú┬¡A-Ponce, B.; Dubrovsky, J.G.; Garay-Arroyo, A.; P ├ú┬┬Rez-Ru ├ ú┬¡Z, R.V.; Kim, S.H.; Acevedo, F.; Pelaz, S.; Alvarez-Buylla, E.R. An AGAMOUS-related MADS-box gene, XAL1 (AGL12), regulates root meristem cell proliferation and flowering transition in Arabidopsis. *Plant Physiology* **2008**, *146*, 1182-1192.
34. Fang, Z.W.; Qi, R.; Li, X.F.; Liu, Z.X. Ectopic expression of FaesAP3 , a Fagopyrum esculentum (Polygonaceae) AP3 orthologous gene rescues stamen development in an Arabidopsis ap3 mutant. *Gene* **2014**, *550*, 200-206.
35. Flanagan, C.A.; Ma, H. Spatially and temporally regulated expression of the MADS-box gene AGL2 in wild-type and mutant arabidopsis flowers. *Plant Molecular Biology* **1994**, *26*, 581-595.
36. Alvarez-Buylla, E.R.; Garc ├¡a-Ponce, B.; Garay-Arroyo, A. Unique and redundant functional domains of APETALA1 and CAULIFLOWER, two recently duplicated Arabidopsis thaliana floral MADS-box genes. *Journal of Experimental Botany* **2006**, *57*, 3099-3107.
37. Goslin, K.; Zheng, B.; Serrano-Mislata, A.; Rae, L.; Ryan, P.T.; Kwa Niewska, K.; Thomson, B.; DS,; Madue O, F.; Wellmer, F. Transcription Factor Interplay between LEAFY and APETALA1/CAULIFLOWER during Floral Initiation. *Plant Physiology* **2017**, *174*, 1097-1109.
38. Gao, R.; Wang, Y.; Gruber, M.Y.; Hannoufa, A. miR156/SPL10 Modulates Lateral Root Development, Branching and Leaf Morphology in Arabidopsis by Silencing AGAMOUS-LIKE 79. *Frontiers in Plant Science* **2017**, *8*, 2226.
39. Kang, I.H.; Steffen, J.G.; Portereiko, M.F.; Lloyd, A.; Drews, G.N. The AGL62 MADS Domain Protein Regulates Cellularization during Endosperm Development in Arabidopsis. *Plant Cell* **2008**, *20*, 635-647.
40. Steffen, J.G.; Kang, I.H.; Portereiko, M.F.; Lloyd, A.; Drews, G.N. AGL61 Interacts with AGL80 and Is Required for Central Cell Development in Arabidopsis. *Plant Physiology* **2008**, *148*, 259-268.
41. Bemer, M.; Woltersarts, M.; Grossniklaus, U.; Angenent, G.C. The MADS Domain Protein DIANA Acts Together with AGAMOUS-LIKE80 to Specify the Central Cell in Arabidopsis Ovules. *Plant Cell* **2008**, *20*, 2088-2101.
42. Portereiko, M.F.; Lloyd, A.; Steffen, J.G.; Punwani, J.A.; Otsuga, D.; Drews, G.N. AGL80 is required for central cell and endosperm development in Arabidopsis. *Plant Cell* **2006**, *18*, 1862-1872.
43. Garay-Arroyo, A.; Ortiz-Moreno, E.; De, l.P.S.â.n.M.; Murphy, A.S.; GarcA-Ponce, B.; Marsch-Mart ├ú ┬¡Nez, N.; De, F.S.; Corvera-Poir, A.; Jaimes-Miranda, F.; Pacheco-Escobedo, M.A. The MADS transcription factor XAL2/AGL14 modulates auxin transport during Arabidopsis root development

by regulating PIN expression. *Embo Journal* **2014**, *32*, 2884-2895.
44. Prez-Ruiz, R.V.; Garca-Ponce, B.; Marsch-Marnez, N.; Ugartechea-Chirino, Y.; Villajuana-Bonequi, M.; DeFolter, S.; Azpeitia, E.; Dvila-Velderrain, J.; Cruz-Snchez, D.; Garay-Arroyo, A. XAANTAL2 (AGL14) Is an Important Component of the Complex Gene Regulatory Network that Underlies Arabidopsis Shoot Apical Meristem Transitions. *Molecular Plant*.
45. Yu, H.; Ito, T.; Wellmer, F.; Meyerowitz, E.M. Repression of AGAMOUS-LIKE 24 is a crucial step in promoting flower development. *Nature Genetics* **2004**, *36*, 157-161.
46. Michaels, S.D.; Ditta, G.; Gustafsonbrown, C.; Pelaz, S.; Yanofsky, M.; Amasino, R.M. AGL24 acts as a promoter of flowering in Arabidopsis and is positively regulated by vernalization. *Plant Journal* **2003**, *33*, 867-874.
47. N, S.n.; R, B.; O, L.; L, B.; C, K.h.; W, G.; L, H. Polycomb-group proteins repress the floral activator AGL19 in the FLC-independent vernalization pathway. *Genes & Development* **2006**, *20*, 1667.
48. Kim, W.; Latrasse, D.; Servet, C.; Zhou, D.X. Arabidopsis histone deacetylase HDA9 regulates flowering time through repression of AGL19. *Biochem Biophys Res Commun* **2013**, *432*, 394-398.
49. Kang, M.; Jin, H.; Noh, Y.; Noh, B. Repression of flowering under a noninductive photoperiod by the HDA9ΓÇÉAGL19ΓÇÉFT module in Arabidopsis. *New Phytologist* **2015**, *206*, 281-294.
50. Gan, Y.; Bernreiter, A.; Filleur, S.; Abram, B.; Forde, B.G. Overexpressing the ANR1 MADS-Box Gene in Transgenic Plants Provides New Insights into its Role in the Nitrate Regulation of Root Development. *Plant & Cell Physiology* **2012**, *53*, 1003-1016.
51. Yu, L.H.; Miao, Z.Q.; Qi, G.F.; Wu, J.; Cai, X.T.; Mao, J.L.; Xiang, C.B. MADS-Box Transcription Factor AGL21 Regulates Lateral Root Development and Responds to Multiple External and Physiological Signals. *Molecular Plant* **2014**, *7*, 1653-1669.
52. Yu, L.H.; Wu, J.; Zhang, Z.S.; Miao, Z.Q.; Zhao, P.X.; Wang, Z.; Xiang, C.B. Arabidopsis MADS-Box Transcription Factor AGL21 Acts as Environmental Surveillance of Seed Germination by Regulating ABI5 Expression. **2017**, *10*, 834-845.
53. Rosloski, S.M.; Singh, A.; Jali, S.S.; Balasubramanian, S.; Weigel, D.; Grbic, V. Functional analysis of splice variant expression of MADS AFFECTING FLOWERING 2 of Arabidopsis thaliana. *Plant Molecular Biology* **2013**, *81*, 57-69.
54. Airoldi, C.A.; Mckay, M.; Davies, B. MAF2 Is Regulated by Temperature-Dependent Splicing and Represses Flowering at Low Temperatures in Parallel with FLM. *Plos One* **2015**, *10*, e0126516.

Supplementary Table 2. Floral organ identity genes and their homologous genes in petunia.

Genes Class	Genes Name	Homologous Gene
Class A	*MC*, *SlMBP20*	*PFG*, *FBP26*, *FBP29*
Class B	*TAP3*, *TM6*, *TPI*, *SlGLO1*	*TM6*, *PMADS1/GP*, *PMADS2*, *FBP1*
Class C	*TAG1*, *TAGL1*	*PMADS3*, *FBP6*, *FBP24*
Class D	*SlMBP3*, *SlMBP22*	*FBP11*, *FBP7*
Class E	*TAGL2*, *TM5*, *SlMADS1*, *SlMBP21*, *SlAGL6*	*FBP2*, *FBP4*, *FBP5*, *FBP9*, *FBP23*, *PMADS4*, *PMADS12*

Supplementary Table 3. Primers for PCR amplification and quantification. All the primers we used were designed by Primer premier 5.0 software.

Primer Names	Sequences (5' →3')
MC-Q-F	AAGTAGCAGAAGCAAGGAGGA
MC-Q-R	CAAGCGATTAGCAAAGAGTGA
MBP20-Q-F	GAAGCTAAAAGAAAATGAGAAGACACA
MBP20-Q-R	GTAAGGTTAGGAAGTTGGTGGTGAG
TAP3-Q-F	TATAAGTCCCTCAATCACGACCA
TAP3-Q-R	GATCATTTAGGCTTTCTCCCATC
TM6-Q-F	CTACAACCATTGCACCCCAAT
TM6-Q-R	CAGGAGAGACGTAGATCACGAGAA
TPI-Q-F	TCTGGGAGGAGACTATGGGATG
TPI-Q-R	TCAGACTGCTTGGCACTGATACTA
GLO1-Q-F	GCTTACTGGAAGAAGATTGTGGG
GLO1-Q-R	CTCATTCTGTTTTTCACGGATACC

Primer Names	Sequences (5' →3')
TAG1-Q-F	ATGAACTTGATGCCAGGGAGT
TAG1-Q-R	GGGGTTGGTCTTGTCTAGGGTA
TAGL1-Q-F	TCGCAATAACTTCCTGCCTGTA
TAGL1-Q-R	AGATGAAGAGCCTTGACCCCA
MBP3-Q-F	ACGAGGCATCAGCAGAATCAG
MBP3-Q-R	GCTGTATTGCACTGTAATCTTGTCC
MBP22-Q-F	CAACTTGGTACTACAAGTAATTCTTCAGC
MBP22-Q-R	AGCTTCTAAATATGCCAAAGGAAAT
TAGL2-Q-F	CAGCAGCAACATCCTCAATCTC
TAGL2-Q-R	CACAGCATCCAACCAGGTATCA
TM5-Q-F	CTTTGTGATGCTGAGGTTGCTC
TM5-Q-R	TTTCCAGTGCTTCTCGTGTTG
MADS1-Q-F	GTGTAGCTGGATTTCCACTTCG
MADS1-Q-R	GCCGCTGCATTCACCTCAT
MBP21-Q-F	AACCTTTCTTTCAACCTCTCCG
MBP21-Q-R	TCCATTAGAGCATCCACCCTG
AGL6-Q-F	GCTTCGTAGAAAGGAGCGTCAT
AGL6-Q-R	GATTTGATTGAGAATGGTGGACATC
MADS-RIN-Q-F	GGAACCCAAACTTCATCAGA
MADS-RIN-Q-R	TTGTCCCAAATCCTCACCTA
TM4-Q -F	AAAATCAGTGGGAAATCAACTCATC
TM4-Q-R	CCTTGCTGCTGTGAAGAACTACC
SlMBP7-Q -F	CCGTGGGAGCAACAGAGTCAT
SlMBP7-Q -R	GGAGGCATCACAGAAGCACTG
CAC-Q-R	ATTGGTGGAAAGTAACATCATCG

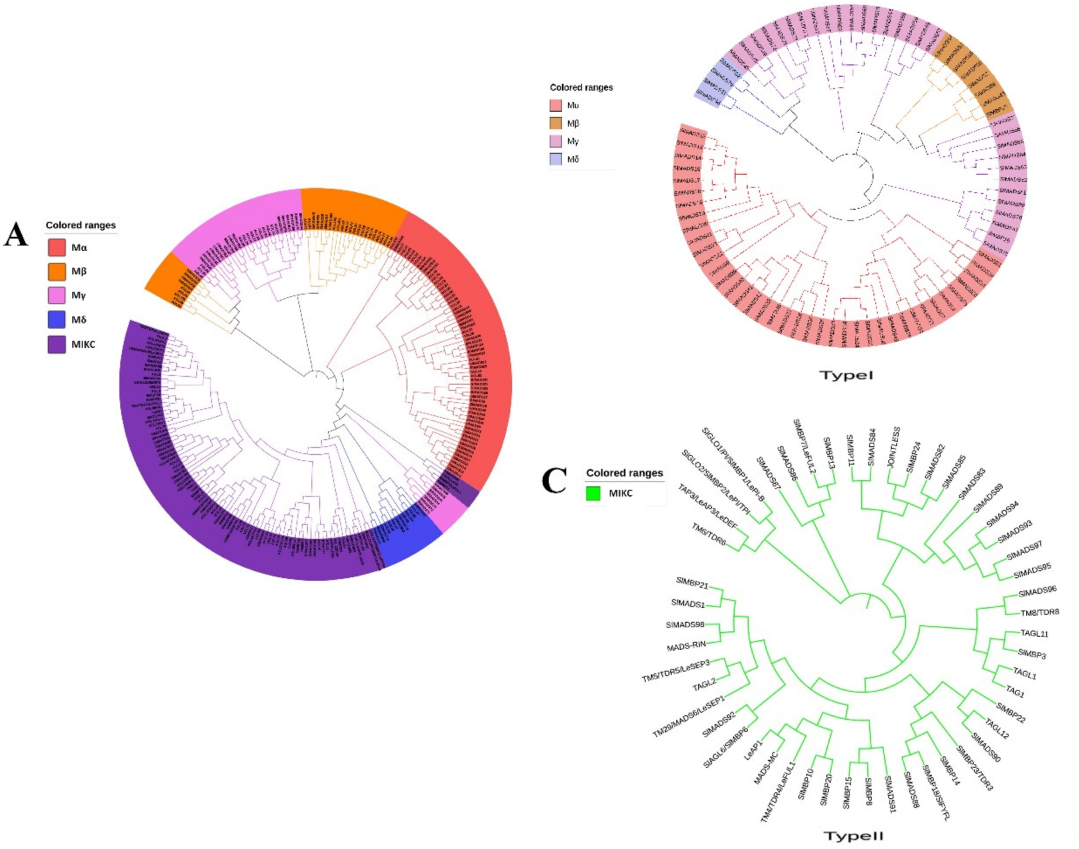

Supplementary Figure 1. Phylogenetic trees. (**A**) Phylogenetic relationship of MADS-box proteins between tomato and Arabidopsis. Five classes are represented by branches of different colors, including Mα (red), Mβ (orange), Mγ (pink), Mδ (blue), and MIKC (purple). Phylogenetic tree of type I (**B**) and type II (**C**) MADS-box domain protein in tomato plants. The phylogenetic tree was

generated using MEGA 5.02 software and the neighbor-joining method with the following parameters: bootstrap analysis of 1,000 replicates, Poisson model, and pairwise deletion.

	Logo	Name	Alt. Name
1.	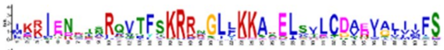	IKRIENKTSRQVTFSKRRNGLLKKAKELSVLCDAEVAJIIFS	MEME-1
2.	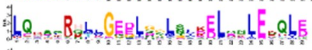	LQRSQRHLLGEDLGGLSLKELZNLEKQLE	MEME-2
3.	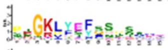	PTGKLYEFSSPSSME	MEME-3
4.	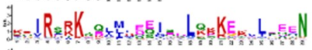	KRIRSRKTQJMLEEISELQKKEKLLEEEN	MEME-4
5.	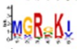	MGRGKI	MEME-5
6.	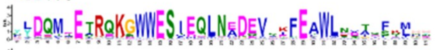	YLDQMEETRQKGWWESIEQLNEDEVSKFEAWLNVTSFKMHH	MEME-6
7.	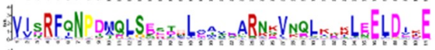	VISRFQNPDMQLSESTHLAAVEARNKVNQLKTKLEELDIRE	MEME-7
8.	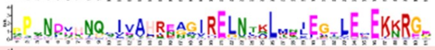	PPNNDVHNQLIVAHREAGIRELNTKLMNJEGELEMEKKRGE	MEME-8
9.	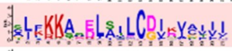	SLFKKASELSILCDIKVAIII	MEME-9
10.	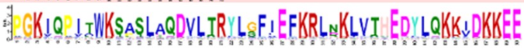	PGKIQPITWKSASLAQDVLTRYLGFIEFKRLNKLVTHEDYLQKKIDKKEE	MEME-10

Supplementary Figure 2. Multilevel consensus sequence among MADS-box genes in tomato were identified by MEME.

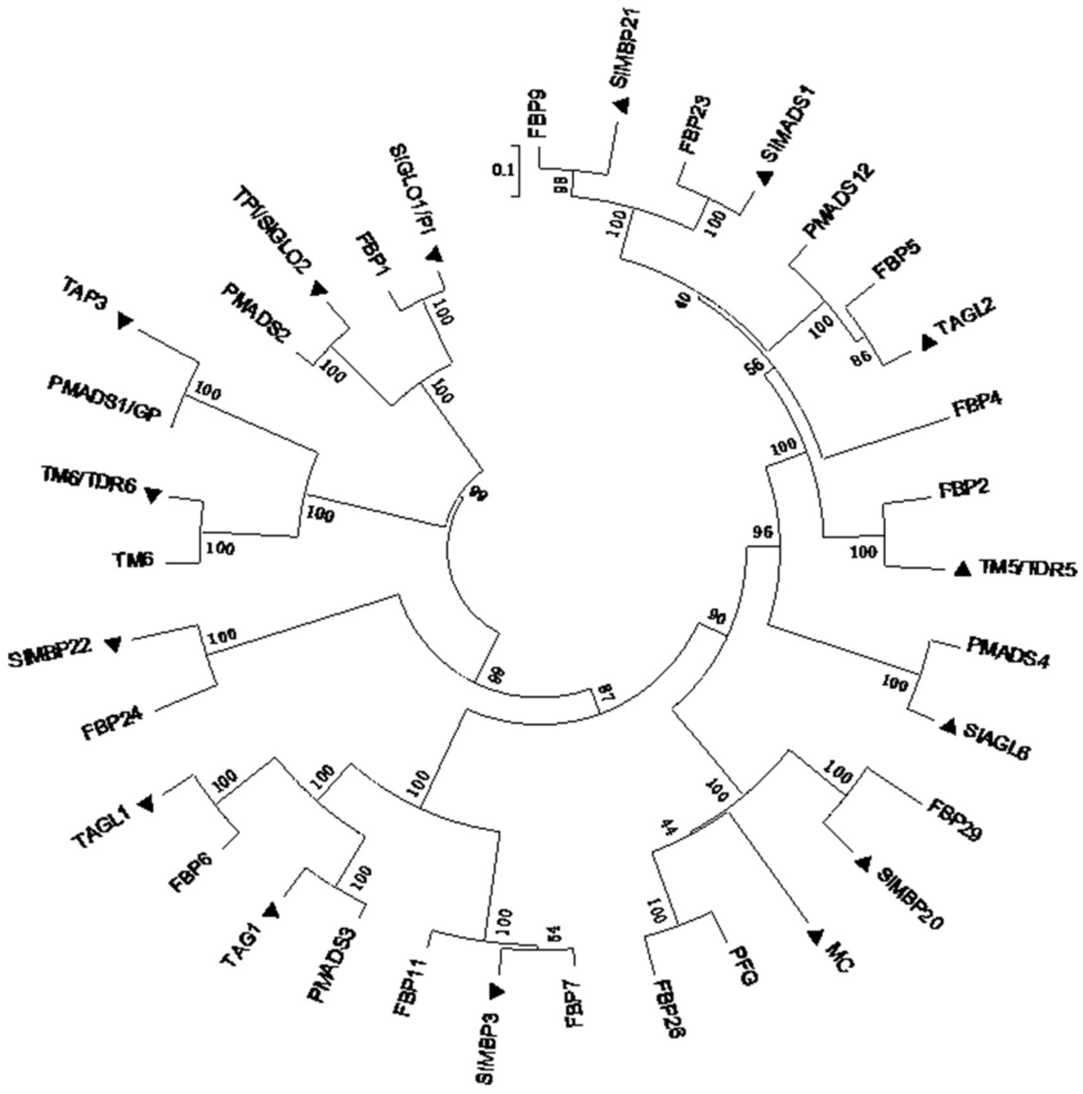

Supplementary Figure 3. Phylogenetic tree of floral organ identity genes in tomato and *Petunia hybrida*. Floral organ identity genes in tomato are marked with black triangles. Accession numbers for other proteins are listed as follows: PFG(AF176782), FBP26(AF176783), FBP29(AF335245), TM6(AF230704), PMADS1/GP(DQ539416), PMADS2(X69947), FBP1(M91190), PMADS3(X72912), FBP6(X68675), FBP24(AF335242), FBP11(X81852), FBP7(X81651), FBP2(M91666), FBP4(AF335234), FBP5(AF335235), FBP9(AF335236), FBP23(AF335241), PMADS4(AB031035), PMADS12(AY370527).

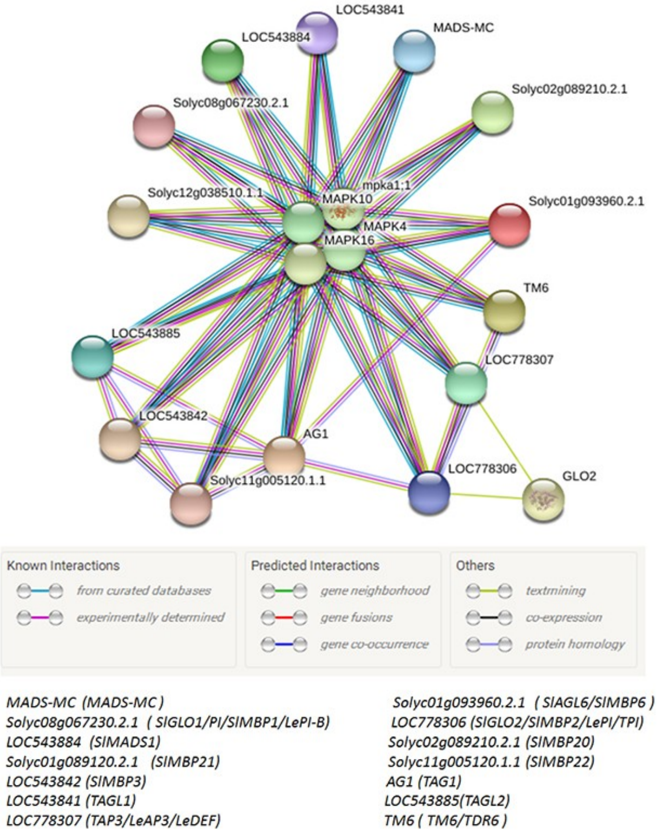

Supplementary Figure 4. Protein-Protein interaction networks of floral organ identity genes. The fifteen tomato MADS-box proteins, involved in floral organ development were analyzed STRING software. Edges represent protein-protein associations.
